# Symptomatic benefit of momelotinib in patients with myelofibrosis: Results from the SIMPLIFY phase III studies

**DOI:** 10.1002/cam4.5799

**Published:** 2023-04-06

**Authors:** Ruben A. Mesa, Stacie Hudgens, Lysbeth Floden, Claire N. Harrison, Jeanne Palmer, Vikas Gupta, Donal P. McLornan, Mary F. McMullin, Jean‐Jaques Kiladjian, Lynda Foltz, Uwe Platzbecker, M. Laura Fox, Adam J. Mead, David M. Ross, Stephen T. Oh, Andrew Perkins, Michael F. Leahy, Samineh Deheshi, Rafe Donahue, Barbara J. Klencke, Srdan Verstovsek

**Affiliations:** ^1^ Atrium Health Wake Forest Baptist Comprehensive Cancer Center, Wake Forest University School of Medicine Winston Salem North Carolina USA; ^2^ Clinical Outcomes Solutions Tucson Arizona USA; ^3^ Guy's and St. Thomas' NHS Foundation Trust London UK; ^4^ Mayo Clinic Phoenix Arizona USA; ^5^ University Health Network, University of Toronto Toronto Ontario Canada; ^6^ Queens University, Belfast City Hospital Trust Belfast UK; ^7^ Hôpital Saint‐Louis, Université de Paris Paris France; ^8^ St Paul's Hospital, University of British Columbia Vancouver British Columbia Canada; ^9^ Leipzig University Hospital Leipzig Germany; ^10^ Hematology Department Hospital Universitario Vall d'Hebron, Experimental Hematology, Vall d'Hebron Institute of Oncology (VHIO), Hospital Universitario Vall d'Hebron, Vall d'Hebron Barcelona Hospital Campus Barcelona Spain; ^11^ MRC Weatherall Institute of Molecular Medicine Oxford UK; ^12^ Flinders Medical Centre and University Adelaide South Australia Australia; ^13^ Washington University School of Medicine St. Louis Missouri USA; ^14^ Alfred Hospital, Monash University Melbourne Victoria Australia; ^15^ University of Western Australia Perth Western Australia Australia; ^16^ Sierra Oncology Plymouth Michigan USA; ^17^ MD Anderson Cancer Center Houston Texas USA

**Keywords:** JAK inhibitor, momelotinib, myelofibrosis, patient‐reported outcomes, symptoms

## Abstract

**Background:**

Myelofibrosis (MF)‐associated constitutional symptoms can severely impact health‐related quality of life. Clinical trials in MF traditionally measure symptom response to treatment as a landmark endpoint of total symptom score (TSS) reduction ≥50% from baseline. However, this dichotomous assessment provides a limited view of clinically relevant symptomatic changes. Herein we evaluated longitudinal change from baseline in TSS over the continuous 24‐week period and individual symptom scores to obtain a more comprehensive understanding of symptom benefits experienced by patients with MF receiving therapy.

**Methods:**

Longitudinal symptom change was evaluated using mixed‐effect model repeated measure (MMRM) methodology with individual item‐level analyses to complement the interpretation of the landmark symptom results in the completed phase III SIMPLIFY studies of momelotinib in MF. MMRM compared mean change in TSS from baseline with Week 24 using data from all patient visits. Generalized estimating equations were used to estimate item‐level odds ratios using multiple predictive imputations for missing data.

**Results:**

Momelotinib and ruxolitinib groups reported similar overall symptom improvements, with a TSS difference of <1.5 points between groups for each post‐baseline visit in SIMPLIFY‐1. In SIMPLIFY‐2, the improvement in TSS observed in momelotinib‐treated patients was consistent with that observed in SIMPLIFY‐1, whereas progressive TSS deterioration was observed with control. Item‐level scores were heterogeneous in both studies. A similar and greater proportion of momelotinib‐treated patients were categorized as “improved” or “stable” compared with control in SIMPLIFY‐1 and SIMPLIFY‐2, respectively. Odds ratios for between‐group comparison ranged from 0.75 to 1.21 in SIMPLIFY‐1, demonstrating similarity in likelihood of symptom improvement. In SIMPLIFY‐2, the likelihood of symptom improvement in each item was higher in the momelotinib arm.

**Conclusions:**

These findings suggest that momelotinib provides clinically relevant symptom benefits in the JAK inhibitor‐naïve and JAK inhibitor‐exposed settings.

## Graphical abstract

Longitudinalmixed‐effect model repeated measure analyses support and expand upon landmarksymptom results in the completed phase III SIMPLIFY studies of momelotinib inMF. Momelotinibprovides consistent and clinically relevant symptom benefits for JAKinhibitor‐naïve and JAK inhibitor‐exposed patients with myelofibrosis.

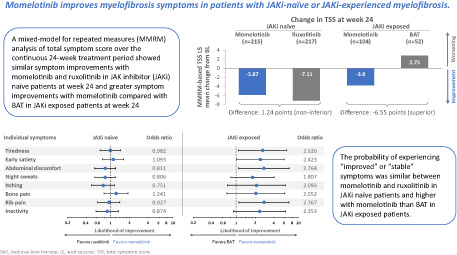

## 1 INTRODUCTION

Myelofibrosis (MF) is a rare myeloproliferative neoplasm characterized by bone marrow fibrosis, constitutional symptoms, anemia, and splenomegaly with an annual incidence of 0.5–1.5 cases per 100,000 individuals in the United States.^1^ Constitutional symptoms triggered by cytokine overproduction cause a significant burden in the physical functioning and health‐related quality of life of patients diagnosed with this chronic and progressive illness. Symptom burden has also been associated with poor prognosis and overall survival.^2^

The manifestation of MF symptoms is heterogeneous because some patients may experience none, some, or all common MF symptoms in differing degrees of severity. Approved JAK inhibitors (i.e., ruxolitinib, fedratinib) are currently recommended as standard of care,^3^ based in part on the ability to improve MF symptom burden,^4^, ^5^, ^6^ but many patients discontinue therapy due to loss of clinical response, intolerability, or adverse toxicity.^7^, ^8^, ^9^

Momelotinib is a potent, selective, orally bioavailable inhibitor of JAK1, JAK2, and activin A receptor type 1 (ACVR1), also known as activin receptor‐like kinase‐2, with a differentiated therapeutic profile in MF, which was examined in the phase III SIMPLIFY trials.^10^, ^11^ In SIMPLIFY‐1, a non‐inferiority study of 432 intermediate‐ and high‐risk JAK inhibitor‐naïve patients with MF randomly assigned 1:1 to momelotinib or ruxolitinib, the primary endpoint of splenic response was met (momelotinib: 26.5% vs. ruxolitinib: 29.5%), but the secondary endpoint of non‐inferiority of total symptom score (TSS) response rate was not met (momelotinib: 28.4% vs. ruxolitinib: 42.2%).^10^ However, in the SIMPLIFY‐2 superiority study of 156 patients previously treated with ruxolitinib and randomly assigned 2:1 to momelotinib or best available therapy (BAT; which was ruxolitinib in 88.5% of patients), a nominally greater proportion of patients assigned to momelotinib achieved symptomatic benefit at Week 24 compared with those randomly assigned to BAT (momelotinib: 26.2% vs. BAT: 5.9%).^11^

Phase III trials evaluating JAK inhibitors in MF have historically defined symptom response as achieving at least a 50% reduction in TSS, measured by the modified Myeloproliferative Neoplasm Symptom Assessment Form (MPN‐SAF) at the end of a 24‐week treatment period compared with baseline.^4^, ^5^, ^6^, ^10^, ^11^ Notably, the study populations in these trials have included both symptomatic and asymptomatic patients. Prior to 2022, studies reflecting the impact of a JAK inhibitor on symptoms in an exclusively symptomatic MF population had not been reported.

Analyses beyond the dichotomous “responder” or “non‐responder” method of evaluating symptom response may further depict the true impact of the disease and treatment effects on patient experiences. A continuous endpoint, such as mean change from baseline as assessed by a mixed‐effect model for repeated measures (MMRM) analysis, has greater statistical power and quantifies the degree of benefit experienced by each patient and may be more discriminatory than the dichotomous response analysis, which defines non‐responders as those with an improvement of less than 50% or achieving no meaningful benefit.

Daily digital collection of patient‐reported outcome (PRO) data allows for longitudinal benefit assessment throughout the duration of the trial rather than landmark data that use scores only from Week 21 through Week 24 for response rate. Furthermore, individual items of the MPN‐SAF are scored collectively to reflect a construct and, therefore, detailed insights into the driving factors that influence treatment‐related changes are lost. For example, some items may have greater clinical importance than others or may be more sensitive to treatment effects.^12^ Changes in TSS could be the result of movement (improvement or worsening) of only a few symptoms but are interpreted as change in the entire construct. Assessing patient‐reported responses on single items can provide meaningful information that may better inform treatment decisions aimed to target specific therapeutic benefits tailored to individual patients in the clinical setting.^13^

Here, we report longitudinal change from baseline of MF domain‐level symptoms using a continuous endpoint based on the MMRM methodology with accompanying individual item‐level analyses to support the interpretation of symptom benefit experienced by patients with MF in each of the 2 SIMPLIFY trials.

## 2 METHODS

### 2.1 Study design and PRO assessments

SIMPLIFY‐1 (NCT01969838) and SIMPLIFY‐2 (NCT02101268) study designs have been previously described.^10^, ^11^ In SIMPLIFY‐1, patients had intermediate‐ or high‐risk primary MF, post‐polycythemia vera (PV) MF, or post‐essential thrombocythemia (ET) MF. Patients with intermediate‐1 risk had symptomatic splenomegaly, hepatomegaly, or anemia (hemoglobin <10.0 g/dL). All patients had palpable splenomegaly ≥5 cm and platelet counts ≥50 × 10^9^/L (≥100 × 10^9^/L if alanine aminotransferase or aspartate aminotransferase ≥2 × upper limit of normal); and must not have received prior JAK inhibitor therapy.^10^ In SIMPLIFY‐2, patients had intermediate‐ or high‐risk primary MF, post‐PV MF, or post‐ET MF. Patients with intermediate‐1 risk had symptomatic splenomegaly or hepatomegaly. All patients had palpable splenomegaly ≥5 cm and no minimum baseline platelet count was required. Patients had been previously treated with ruxolitinib for ≥28 days and had experienced hematologic toxicity while receiving ruxolitinib, and a washout period from prior ruxolitinib treatment was prohibited for patients actively receiving ruxolitinib as they entered study screening.^11^

The modified MPN‐SAF version 2 is an eight‐item patient‐reported questionnaire developed to assess symptom burden in patients with MPNs. All items were assessed by patients as the “worst incidence” in the prior 24 h using a 0–10 numeric rating scale, with 0 corresponding to “absent” and 10 corresponding to “worst imaginable.” The daily TSS was calculated as the sum of seven of the eight items (excluding the eighth item, “inactivity”) for a score ranging from 0 to 70, with higher TSS indicating greater severity. Baseline TSS was calculated as the average of daily TSS of the 7 days prior to randomization. Upon randomization, the MPN‐SAF was administered daily for the first 24 weeks of both the SIMPLIFY‐1 and SIMPLIFY‐2 clinical trials. For the prespecified analysis at Week 24, TSS was calculated as the average score from the prior 28‐day period and compared with baseline.

### 2.2 Statistical analysis, general considerations

The analytic population was defined as patients with PRO assessments at baseline and at least one post‐baseline assessment at any time point within the intention‐to‐treat population. All analyses were performed using SAS version 9.4.

Descriptive statistics were used to characterize patient demographics, clinical characteristics, and PRO instrument completion rates.^10^, ^11^ The SIMPLIFY‐1 and SIMPLIFY‐2 trials included a distribution of symptomatic (defined post hoc as MPN‐SAF TSS ≥10) and asymptomatic (MPN‐SAF TSS <10) patients.

### 2.3 Change in TSS on the modified MPN‐SAF V2.0: Derivation of a non‐inferiority margin

As a double‐blind, head‐to‐head comparison of two active JAK inhibitors, momelotinib versus ruxolitinib, a prespecified objective of SIMPLIFY‐1 was to demonstrate non‐inferiority for TSS response at Week 24. In SIMPLIFY‐2, the open‐label comparison of momelotinib to BAT in previously JAK inhibitor‐treated patients, a prespecified objective was to demonstrate superiority for TSS at Week 24.

Here, the method for deriving the between‐groups non‐inferiority margin in SIMPLIFY‐1 considers the preserved fraction of the variability closest to estimating a null effect MPN‐SAF TSS change score. In this case, the upper bound of the 95% CI for the overall change score in the population at Week 24 is preserved. Results were presented for preserved fractions of 50%, 75%, and 90%. To support this interpretation, the magnitude of the effect size differences in MPN‐SAF TSS was presented using Cohen's *d* thresholds: ≥0.20, ≥0.50, and ≥0.80 (small, moderate, and large), with the hypothesis that the estimate does not exceed a moderate effect (≥0.50; Cohen's *d*).^14^

$$\left(1-M1\right)\times \text{ULCI}$$

where *M*1 is the preserved fraction and ULCI is the upper limit confidence interval TSS pooled change score.

The within‐patient‐group non‐inferiority margin for SIMPLIFY‐1 in this study used two metrics: (1) the anchor‐based meaningful change threshold (MCT) derived using treatment agnostic trial data^15^ and (2) a sensitivity value of the upper limit CI of the MCT. The derived MCT provides a parameter for determining the degree to which the within‐patient change from baseline at Week 24 exceeds an amount meaningful to patients within the trial. The Patient Global Impression of Change (PGIC) anchor was used to derive the MCT in SIMPLIFY‐1.^15^ Responses to the PGIC were anchored to the TSS change from baseline for each category of change. An MCT was chosen to ensure no overlap in the range of TSS in the “no change” group compared with the “minimally improved” group. The upper limit confidence interval (ULCI) for the “within‐group non‐inferiority margin” represents the ULCI of the absolute TSS change from baseline in the minimally improved group for the overall and symptomatic populations, respectively, leading to the selection of eight points as the MCT.

### 2.4 Mixed‐effect model for repeated measure

The longitudinal MMRM model was used to evaluate the adjusted treatment group mean differences in SIMPLIFY‐1 and SIMPLIFY‐2. The differences in mean change of MPN‐SAF TSS from baseline across each planned assessment (i.e., every‐4 week interval up to Week 24) were tested using an MMRM model with treatment, time, treatment‐by‐time interaction, age, race, and baseline MPN‐SAF TSS included as fixed and random covariates. The restricted maximum likelihood estimation was implemented with an unstructured covariance matrix shared across treatment groups for modeling the within‐patient errors.

Least squares (LS) means, corresponding standard errors, and 95% two‐sided CIs were determined for the within‐group change for all time points. For the between‐treatment group comparison, the difference in LS means, corresponding standard errors, 95% two‐sided CI, and *p‐*value were reported. Graphic representation of change from baseline was presented using cumulative distribution function (CDF) curves.

### 2.5 Change from baseline in individual symptoms and categorical responder analysis

Descriptive analyses of the individual MPN‐SAF TSS items include the absolute proportion of patients assigned to each response category at baseline and at Week 24 based on the average of their reported scores (none: 0 points; mild: 1–3 points; moderate: 4–6 points; severe: 7–9 points; very severe: 10 points). Missing responses were not included in the denominator reporting proportions of responses.

The proportion of patients experiencing change (e.g., improved, stable, declined/worsened) was also reported at Week 24. “Declined/worsened” was defined as an increase of at least two points, “stable” as a change of at most one point, and “improved” as a decrease of at least two points.

### 2.6 Generalized estimation equations

Generalized estimating equations were used to model categorical change on the individual symptom items of the MPN‐SAF v2.0 to estimate the treatment‐related difference in the odds of “improved” versus “stable or declined/worsened.” Models included fixed effects of treatment, time point, treatment‐by‐time point interaction, and stratification variables, along with an R‐side random patient effect. Results were accompanied by graphic presentations for each statistical test conducted including forest plots of odds ratios for experiencing meaningful change in the treatment group versus placebo with 95% CIs around each odds ratio for each symptom item.

## 3 RESULTS

### 3.1 Study population

Overall, patient demographics and clinical characteristics were similar across treatment groups for both studies (Tables S1 and S2). The median TSS in the SIMPLIFY‐1 overall population was 17.0 (momelotinib: 17.4; ruxolitinib: 16.4), whereas in the symptomatic population it was 23.0 (momelotinib: 23.5; ruxolitinib: 21.9). Baseline TSS was available for 427 of the 432 patients enrolled in SIMPLIFY‐1; 135 patients had a baseline TSS <10 points (68 patients in the momelotinib arm and 67 patients in the ruxolitinib arm). The median TSS in the overall SIMPLIFY‐2 population was 15.6 (momelotinib: 15.6; BAT: 15.9), whereas in the symptomatic population, the median TSS was 22.4 (momelotinib: 21.6; BAT: 24.1). Of the 152 patients enrolled in SIMPLIFY‐2, 51 patients had a baseline TSS <10 (32 patients in the momelotinib arm and 19 patients in the BAT arm).

### 3.2 Non‐inferiority margin (SIMPLIFY‐1)

The derived non‐inferiority margin for SIMPLIFY‐1 is presented in Table 1. Non‐inferiority margins were then applied to support the interpretation of clinical meaningfulness for the between‐treatment groups–and within‐patient groups–change from baseline in the MMRM analyses. The non‐inferiority margin for the between‐treatment group comparison was set using the ULCI of the absolute TSS change from baseline at Week 24 in the pooled momelotinib and ruxolitinib groups (overall population: −5.80; symptomatic population: −8.28).

**TABLE 1:** SIMPLIFY‐1 non‐inferiority margins.

SIMPLIFY‐1 non‐inferiority margin				
Non‐inferiority margin (between groups)		Fraction of the margin		
Population	ULCI (absolute TSS population change from baseline at Week 24)	50%	75%	90%
Overall	−5.80	−2.90	−1.45	−0.58
Symptomatic	−8.28	−4.14	−2.07	−0.83
Non‐inferiority margin (within groups)				
Population	MCT value^a^	ULCI (PGIC minimally improved group anchored to absolute TSS change from baseline at Week 24)		
Overall	−8.0	−3.38		
Symptomatic	−8.0	−5.16		

Abbreviations: MCT, meaningful change threshold; PGIC, Patient Global Impression of Change; TSS, total symptom score; ULCI, upper limit confidence interval.

^a^ The non‐inferiority margin for the within‐patient group used the ULCI of the MCT, which was derived to be 8 points. The MCT of 8 points was determined using an anchor‐based method as previously described.^15^

### 3.3 Mixed‐effect model for repeated measure analyses

Change in total scores on the modified MPN‐SAF v2.0 by treatment is shown in Table 2. In SIMPLIFY‐1, the actual LS mean progressively decreased from Week 1 to Week 24, indicating that both momelotinib and ruxolitinib groups reported an improvement in MF‐associated symptoms. Specifically, in the overall population, LS mean change from baseline was −5.87 and −7.11 for the momelotinib and ruxolitinib groups, respectively. This exceeded the sensitivity non‐inferiority margin of −3.38 and indicates that a minimal improvement (i.e., a 1‐category improvement) was observed for most patients. Similar results were observed for the symptomatic population, where the LS mean change at Week 24 for the momelotinib treatment group was −8.12 versus −9.39 for the ruxolitinib group. This exceeded the sensitivity margin of −5.16 as well as the MCT non‐inferiority margin of −8.0, indicating meaningful improvement was achieved by most patients in the symptomatic population in both arms.

**TABLE 2:** MMRM‐based TSS change from baseline.

	Primary model^a^			
	Overall population		Symptomatic population (baseline MPN‐SAF TSS ≥10)	
SIMPLIFY‐1	MMB (*n* = 215)	RUX (*n* = 217)	MMB (*n* = 145)	RUX (*n* = 147)
(non‐inferiority)				
Baseline				
LS mean (SE)	18.98 (1.73)	17.48 (1.74)	25.13 (1.92)	23.11 (1.92)
Week 24				
Overall change				
LS mean (SE)	−5.87 (0.93)	−7.11 (0.91)	−8.12 (1.22)	−9.39 (1.20)
Difference from ruxolitinib				
LS mean (SE)	1.24 (0.83)		1.26 (1.11)	
95% CI	−0.40, 2.88		−0.92, 3.45	
SIMPLIFY‐2	MMB (*n* = 104)	BAT (*n* = 52)	MMB (*n* = 75)	BAT (*n* = 35)
(superiority)				
Baseline				
LS mean (SE)	16.71 (2.09)	19.84 (2.69)	23.13 (2.65)	28.82 (3.34)
Week 24				
Overall change				
LS mean (SE)	−3.80 (1.38)	2.75 (1.87)	−5.92 (2.05)	2.45 (2.76)
Difference from BAT				
LS mean (SE)	−6.55 (1.99)		−8.37 (2.75)	
95% CI	−10.48, −2.61		−13.83, −2.91	

Abbreviations: BAT, best available therapy; LS, least squares; MPN‐SAF, Myeloproliferative Neoplasm Symptom Assessment Form; MMB, momelotinib; MMRM, mixed‐effect model for repeated measure; RUX, ruxolitinib; SE, standard error; TSS, total symptom score.

^a^ The primary model includes treatment, time, treatment × time, age, race.

Comparing between‐treatment groups, a slightly greater reduction in the Week 24 LS mean change scores from baseline was observed in the ruxolitinib treatment group compared with the momelotinib treatment group (a difference of 1.24 points in the overall population). The differences in the overall population did not exceed the ‐2.90 non‐inferiority 50% fraction of the margin and was near the −1.45 non‐inferiority 75% fraction of the margin. Similar results were observed in the symptomatic population, where a difference of 1.26 points did not exceed the −4.14 non‐inferiority 50% fraction of the margin or the −2.07 non‐inferiority 75% fraction of the margin (Table 1).

In SIMPLIFY‐2, the LS mean scores progressively decreased from baseline to Week 24 for the momelotinib group, and progressively increased in the BAT group. Specifically, in the overall population, LS mean change from baseline was −3.80 at Week 24 in the momelotinib group, and +2.75 at Week 24 in the BAT group (Table 2). In the symptomatic population, the LS mean change from baseline was −5.92 at Week 24 in the momelotinib group, and +2.45 at Week 24 in the BAT group (Table 2).

**FIGURE 1:**
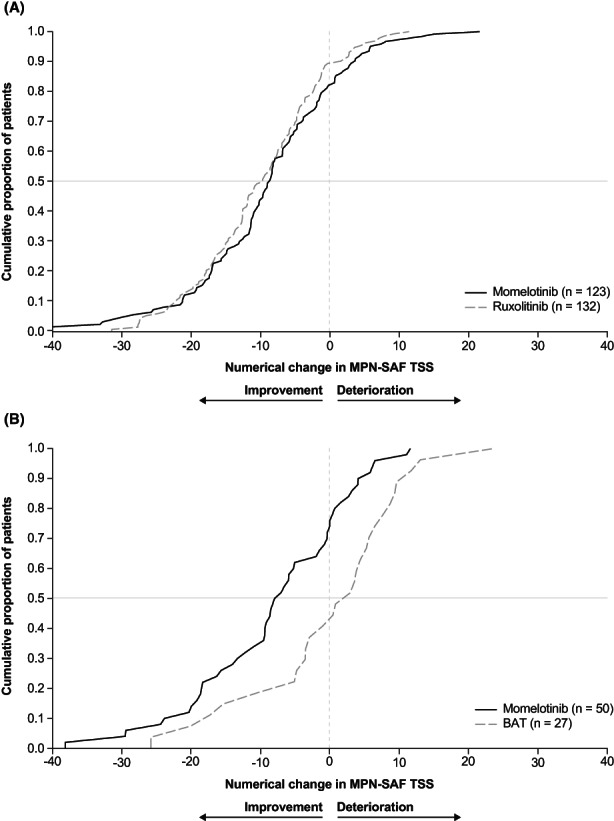
Cumulative distribution function of absolute change in MPN‐SAF TSS from baseline to Week 24 in the (A) SIMPLIFY‐1 symptomatic population and (B) SIMPLIFY‐2 symptomatic population. BAT, best available therapy; MPN‐SAF, Myeloproliferative Neoplasm Symptom Assessment Form; TSS, total symptom score.

Cumulative distribution function of change in MPN‐SAF TSS from baseline at Week 24 by treatment in the symptomatic populations of SIMPLIFY‐1 and SIMPLIFY‐2 are shown in Figure 1. In SIMPLIFY‐1, the CDF curves for momelotinib and ruxolitinib are nearly overlapping, demonstrating that the distribution of absolute change in TSS is similar in the momelotinib and ruxolitinib groups (Figure 1A). In the SIMPLIFY‐2 CDF curve, momelotinib shows a greater proportion of patients in the improvement levels of change (Figure 1B). Cumulative distribution function of change in MPN‐SAF TSS from baseline at Week 24 in the overall populations of SIMPLIFY‐1 and SIMPLIFY‐2 were generally similar to those of the symptomatic population and are shown in Figure S1.

### 3.4 Individual item analysis

Distribution of individual item scores at baseline was examined (Figure S2). Mean scores for each item demonstrated variability in severity in the overall and symptomatic populations of both SIMPLIFY‐1 and SIMPLIFY‐2. In the SIMPLIFY‐1 momelotinib group, for example, 38% reported no or mild tiredness, and 79% reported no or mild itching. Tiredness remained the most severe and prevalent item reported at baseline in both treatment groups. Consistent with other items in SIMPLIFY‐1, patients were more likely to report severe or very severe tiredness scores at baseline in the momelotinib group than in the ruxolitinib group. In SIMPLIFY‐2, patients were more likely to report severe or very severe tiredness scores at baseline in the BAT group. Itching was the least prevalent item reported across both studies at baseline in either treatment arms. Similar results were observed in the symptomatic population (Figure S2).

**FIGURE 2:**
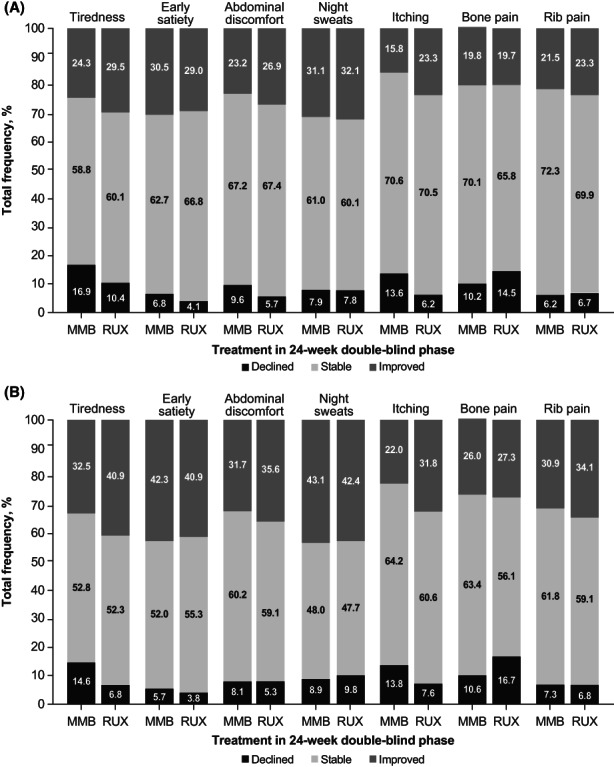
Percent improved, stable, or declined/worsened on MPN‐SAF TSS items by treatment group in SIMPLIFY‐1 (A) overall and (B) symptomatic populations. MMB, momelotinib; MPN‐SAF, Myeloproliferative Neoplasm Symptom Assessment Form; RUX, ruxolitinib; TSS, total symptom score.

**FIGURE 3:**
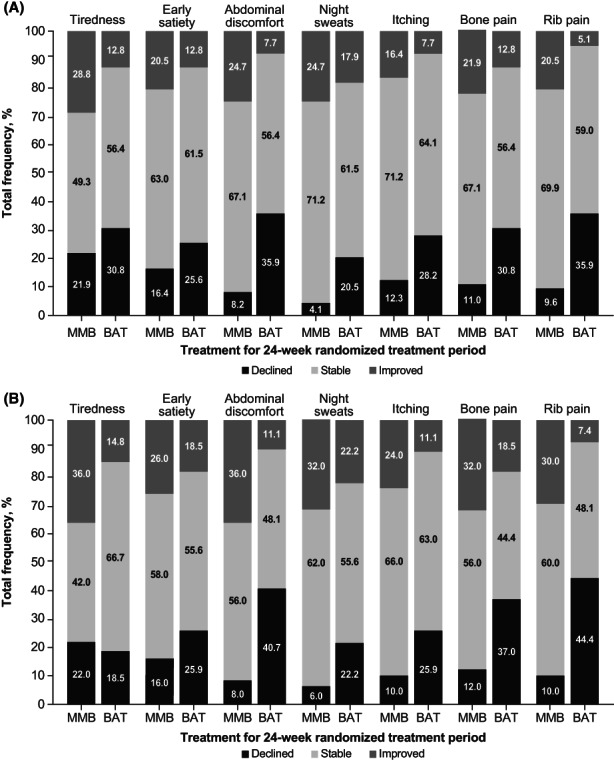
Percent improved, stable, or declined/worsened on MPN‐SAF TSS items by treatment group in SIMPLIFY‐2 (A) overall and (B) symptomatic populations. BAT, best available therapy; MMB, momelotinib; MPN‐SAF, Myeloproliferative Neoplasm Symptom Assessment Form; RUX, ruxolitinib; TSS, total symptom score.

**FIGURE 4:**
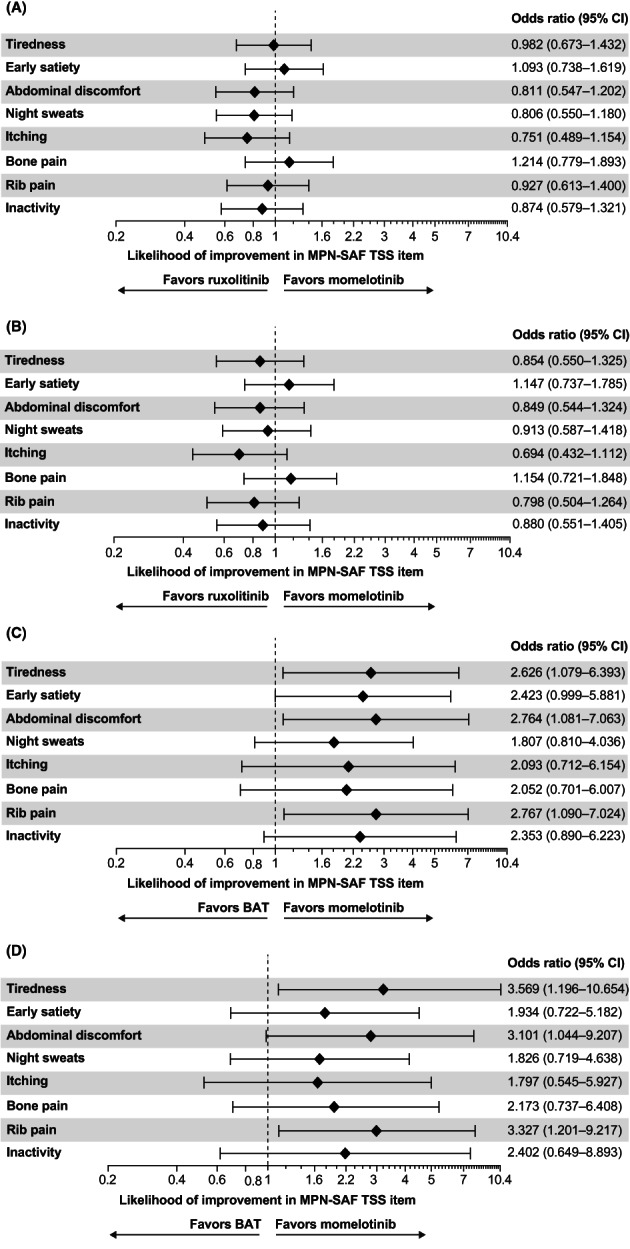
Likelihood of improvement for the MPN‐SAF TSS items in the (A) SIMPLIFY‐1 overall population, (B) SIMPLIFY‐1 symptomatic population, (C) SIMPLIFY‐2 overall population, and (D) SIMPLIFY‐2 symptomatic population. BAT, best available therapy; MPN‐SAF, Myeloproliferative Neoplasm Symptom Assessment Form; TSS, total symptom score.

The majority of patients in SIMPLIFY‐1 and SIMPLIFY‐2 experienced stability or improvement (i.e., a change of at most one point or an improvement of at least two points, respectively, from baseline to Week 24) in both the overall and symptomatic populations (Figures 2 and 3). In the overall population in SIMPLIFY‐1, rates of symptom improvement and stability were similar in both the momelotinib and ruxolitinib arms. Rates of symptom decline/worsening were generally low, particularly for early satiety, abdominal discomfort, and rib pain (below 8% in both groups). In general, symptomatic patients in SIMPLIFY‐1 experienced higher rates of symptom improvement than the overall population, with rates of declined/worsened remaining low. In SIMPLIFY‐2, patients in the overall and symptomatic populations treated with momelotinib experienced higher rates of symptom improvement and stability, but lower rates of symptom decline/worsening than patients who received BAT. The likelihood of experiencing any improvement over the course of the study in SIMPLIFY‐1 was roughly equivalent in the momelotinib and ruxolitinib arms with variability in the observed likelihood estimates (Figure 4). More specifically, the criterion value for equivalence is an odds ratio = 1.0. In the observed outcomes, the CIs suggest that additional covariates may impact these outcomes because the CIs for each item consistently fall above and below this value. In SIMPLIFY‐2, the likelihood of experiencing improvement in each symptom was higher in the momelotinib arm. Forest plots for the likelihood of improvement are shown in Figure 4.

## 4 DISCUSSION

Here, we employed MMRM methodology with accompanying individual item‐level analyses using a continuous endpoint to examine longitudinal changes in MF domain‐level symptoms in each of the phase III SIMPLIFY trials of momelotinib. The benefits of using the MMRM approach are that it (1) estimates TSS treatment effect as a continuous variable and provides greater statistical power and clinically relevant granularity, (2) incorporates longitudinal data from every 4‐week period leading up to Week 24 (not just the terminal 4 weeks), (3) does not automatically assign a non‐responder status to patients with missing data but rather models the outcome at each time point where data are present, and (4) quantifies the overall degree of benefit experienced by each patient. Thus, this MMRM model provides an improved understanding of symptom response and the degree of benefit experienced by each patient, when compared with the dichotomous landmark symptom response endpoint traditionally used in MF clinical trials. The benefit of using individual item‐level data is to comprehensively inform the baseline severity and response to treatment over time of symptoms that compose the TSS. Future technological advances, such as the development of an application for patients to self‐monitor their symptoms, may be useful for clinical practice. Findings from these MMRM and individual item analyses demonstrate comparable symptom improvement on the MPN‐SAF TSS between the momelotinib and ruxolitinib treatment arms in SIMPLIFY‐1 and greater improvement in the momelotinib group compared with the BAT group in SIMPLIFY‐2, supporting the clinical relevance of symptom benefits with momelotinib for JAK inhibitor‐naïve and JAK inhibitor‐exposed patients with MF.

Several design nuances in the SIMPLIFY‐1 trial limited the interpretability of the symptom response outcome. First, the prespecified TSS endpoint was based on a 50%‐responder definition, with the non‐inferiority margin based on historical data of a six‐item score from the MPN‐SAF to measure TSS, whereas the seven‐item score from the modified MPN‐SAF v2.0 was used to determine responder rate in SIMPLIFY‐1. The six‐item score does not include tiredness, typically the most severe and prevalent item. The SIMPLIFY‐1 secondary endpoint of TSS response rate at Week 24 failed to demonstrate non‐inferiority for momelotinib versus ruxolitinib. Second, baseline severity on the MPN‐SAF TSS was not an inclusion criterion in SIMPLIFY‐1, which led to a substantial number of asymptomatic patients entering the trial. For those patients, an a priori maintenance hypothesis was not considered. This may also have resulted in variations in symptom improvement between the momelotinib and ruxolitinib arms because (1) the 50% improvement threshold required for response may be achieved by vastly different absolute scores when patients with high and low baseline TSS are analyzed alongside each other; and (2) TSS was not a stratification factor, which resulted in a numerically higher mean baseline TSS in patients in the momelotinib arm than the ruxolitinib arm. This was evidenced by our results herein showing that the momelotinib and ruxolitinib groups achieved very similar absolute change in TSS from baseline when excluding patients with very low TSS (<10) at baseline. Additionally, missing data may have also contributed to the variation in symptom improvement between the momelotinib and ruxolitinib arms. More patients in the momelotinib arm discontinued treatment early due to low‐grade adverse events, which resulted in an imbalance in early discontinuation rates between the treatment arms (momelotinib: 15%; ruxolitinib: 6%). The SIMPLIFY‐1 study protocol included a ruxolitinib‐oriented dose modification schema, which likely led to higher rates of dose reductions and interruptions, but lower rates of early discontinuation due to low‐grade adverse events with ruxolitinib. Because patients who discontinued prior to Week 24 were considered non‐responders in the TSS analysis, this contributed to a lower TSS response rate at Week 24 in the momelotinib arm. Furthermore, change from baseline was not considered in the primary or key secondary endpoint strategy. Scores were dichotomized into responder categories rather than using the full score variability (e.g., heterogeneity of variance was not assessed).

There were also several design nuances in the SIMPLIFY‐2 study that limited the interpretability of the PROs. Specifically, the only prespecified MPN‐SAF TSS endpoint performed was based on a 50%‐responder definition; the new analyses in this report augment this standard TSS response definition. Also, SIMPLIFY‐2 was an open‐label study, which could be regarded with more bias than a blinded trial. However, to address this potential bias, multiple item and domain level analyses were conducted and the impact of the open‐label design on symptomatic benefit appeared negligible, as observed in similar studies.^16^ Specifically, similar completion rates were observed and the magnitude of benefit observed in the momelotinib group was substantial compared with that in BAT as shown by the higher symptomatic responder rates, MMRM, and individual item analyses.

In both SIMPLIFY‐1 and SIMPLIFY‐2, the items on the MPN‐SAF TSS were assumed unidimensional in the scoring; however, the face validity review demonstrates heterogeneity across the instrument. Furthermore, the studies were not powered to assess differences in individual items of TSS, which vary in prevalence. One limitation of the MMRM model is that it assumes a missing observation is equally likely to be a response or a non‐response, regardless of the health status of the patient, whereas the dichotomous approach assumes that all patients with missing data are not responders. However, it is not possible to confirm whether MMRM modeling yielded results that more closely reflect the true treatment differences than the dichotomous approach.

Beyond these limitations, evidence from the SIMPLIFY studies demonstrates that momelotinib provides comprehensive improvements in symptoms with a similar magnitude across trials, reduction in spleen size, decreased anemia, and reduced transfusions.^10^, ^11^ The efficacy of momelotinib is consistent with inhibition of JAK1 and JAK2, whereas the inhibitory function of ACVR1 is unique among the JAK inhibitor class, leading to differentiated pro‐erythropoietic activity. Individual item analyses further support the anemia‐ and spleen‐associated benefits of momelotinib through improvement in fatigue, abdominal pain, early satiety, and pain under ribs on the left side.

Findings from the SIMPLIFY trials support the demonstration of symptomatic improvement in terms of statistical results and clinically meaningful interpretation. Metrics for deriving between‐ and within‐patient analysis contextualize the TSS findings providing further confidence in the symptomatic benefit of momelotinib. Pertinent findings from the analyses described in this report were considered in the design of the PRO‐specific statistical analysis plan for the MOMENTUM phase III study (NCT04173494) to ensure appropriate interpretation of the symptom data arising from that study.

Overall, results from SIMPLIFY‐1 illustrate that the momelotinib‐treated patients had similar symptom benefits to ruxolitinib treatment groups for both the overall and symptomatic populations. Differences between treatments were small relative to the derived non‐inferiority margin. The magnitude of improvement for each item was similar, with some symptoms favoring momelotinib treatment and others favoring ruxolitinib. Results from SIMPLIFY‐2 support the finding that momelotinib significantly improves MF symptoms compared with the BAT group, where 88.5% of patients were treated with ruxolitinib. The magnitude of symptom response for patients treated with momelotinib was similar in both SIMPLIFY studies, demonstrating that momelotinib provides robust symptom benefit in JAK inhibitor‐naïve patients and in those with prior JAK inhibitor treatment. This study, together with other evidence from SIMPLIFY‐1 and SIMPLIFY‐2, demonstrates that momelotinib addresses the key hallmark features of MF and the unmet medical needs for patients with MF.

## AUTHOR CONTRIBUTIONS

Data collection: Ruben A. Mesa, Claire N. Harrison, Jeanne Palmer, Vikas Gupta, Donal P. McLornan, Mary F. McMullin, Jean‐Jaques Kiladjian, Lynda Foltz, Uwe Platzbecker, M. Laura Fox, Adam J. Mead, David M. Ross, Stephen T. Oh, Andrew Perkins, Michael F. Leahy, and Srdan Verstovsek. Study conceptualization and data analysis: Stacie Hudgens, Lysbeth Floden, Samineh Deheshi, Rafe Donahue, Barbara Klencke, Ruben Mesa, Srdan Verstovsek. Data interpretation and manuscript revisions and approval for submission: All authors.

## FUNDING STATEMENT

SIMPLIFY‐1 and SIMPLIFY‐2 were sponsored by Gilead Sciences. This analysis was sponsored by Sierra Oncology, a GSK company.

## CONFLICT OF INTEREST STATEMENT

Ruben A. Mesa has received research funding from AbbVie, Celgene/Bristol Myers Squibb, CTI BioPharma, Constellation, Genentech, Incyte, Promedior, Samus, and Mays Cancer Center P30 Cancer Center Support Grant from National Cancer Institutes (CA054174) and has served as a consultant for Constellation, La Jolla, Novartis, and Sierra Oncology. Stacie Hudgens and Libby Floden are employees of Clinical Outcomes Solutions, which received funding for the statistical analysis. Claire N. Harrison has received research funding from Bristol Myers Squibb, Constellation, and Novartis; has served as a speaker for AOP, AbbVie, Celgene/Bristol Myers Squibb, Constellation, CTI BioPharma, Janssen, Novartis, and Sierra Oncology; has consulted for AOP, AbbVie, CTI BioPharma, Galecto, Generon, Roche, and Sierra Oncology; has served on a data safety monitoring board or advisory board for Galecto; and has received meeting and/or travel support from Bristol Myers Squibb and Novartis. Jeanne Palmer has received research funding from CTI BioPharma, Incyte, PharmaEssentia, Protagonist Therapeutics, and Sierra Oncology and has served as an advisor for CTI BioPharma, PharmaEssentia, and Sierra Oncology. Vikas Gupta has served as a consultant for AbbVie, Celgene/Bristol Myers Squibb, Constellation, Novartis, Pfizer, and Sierra Oncology; has served as a speaker for Celgene/Bristol Myers Squibb, Constellation, and Novartis; and has served on a data safety monitoring board or advisory board for AbbVie, Celgene/Bristol Myers Squibb, Roche, and Pfizer. Donal P. McLornan has served as a speaker for AbbVie, Celgene/Bristol Myers Squibb, Jazz Pharmaceuticals, and Novartis; has received meeting and/or travel support from Jazz; and served on a data safety monitoring board or advisory board for Jazz. Mary F. McMullin has served as a speaker for AOC, AbbVie, Incyte, Novartis, and Pfizer and has received consulting fees from AbbVie, Bristol Myers Squibb, CTI BioPharma, Novartis, and Sierra Oncology. Jean‐Jaques Kiladjian has served on advisory boards or as a consultant for AOP Orphan Pharmaceuticals, Celgene/Bristol Myers Squibb, and Novartis. Lynda Foltz has served as a speaker for Celgene/Bristol Myers Squibb and Novartis Canada and served on a data safety monitoring board or advisory board for Celgene/Bristol Myers Squibb, Incite, and Novartis Canada. Uwe Platzbecker has served as a consultant for AbbVie, Celgene/Bristol Myers Squibb, Janssen, and Novartis; has served as a speaker for Amgen, Jazz, and Takeda; and has served on a data safety monitoring board or advisory board for AbbVie and Novartis. M. Laura Fox has served as a speaker for Novartis and Sierra Oncology; has received meeting and/or travel support from MDS, Novartis, and Pfizer; and served on a data safety monitoring board or advisory board for Bristol Myers Squibb, Novartis, and Sierra Oncology. Adam J. Mead has served as a consultant for AbbVie, Celgene/Bristol Myers Squibb, Galecto, Gilead, Incyte, Karyopharm, Novartis, Sensyne Health, and Sierra Oncology; has served as a speaker, received meeting and/or travel support; and participated on a data safety monitoring board or advisory board for Celgene/Bristol Myers Squibb. David M. Ross has served on advisory boards and as a speaker for Celgene/Bristol Myers Squibb, Imago, Keros, and Novartis; has received meeting and/or travel support from Novartis; has served as a consultant for Keros and Novartis; and has had an unpaid leadership role in the Australasian Leukaemia and Lymphoma Group Specialist Advisory Committee. Stephen T. Oh has served as a consultant for AbbVie, Celgene/Bristol Myers Squibb, Blueprint Medicines, Constellation, CTI BioPharma, Disc Medicine, Kartos Therapeutics, PharmaEssentia, and Sierra Oncology. Andrew Perkins has served as a speaker, received meeting and/or travel support, and served on a data safety monitoring board or advisory board for Novartis. Michael F. Leahy has nothing to disclose. Samineh Deheshi, Rafe Donahue, and Barbara J. Klencke are employees of Sierra Oncology, a GSK company. Srdan Verstovsek has received research funding from AstraZeneca, Blueprint Medicines, Celgene/Bristol Myers Squibb, CTI BioPharma, Genentech, Gilead, Incyte, Italfarmaco, Novartis, NS Pharma, Pharma Essentia, Promedior, Protagonist Therapeutics, Roche, and Sierra Oncology and has served as a consultant for Celgene/Bristol Myers Squibb, Incyte, Novartis, and Sierra Oncology.

## ETHICS STATEMENT

SIMPLIFY‐1 and SIMPLIFY‐2 were approved by institutional review boards or independent ethics committees.

## PATIENT CONSENT STATEMENT

All patients who participated in SIMPLIFY‐1 and SIMPLIFY‐2 provided written informed consent.

## CLINICAL TRIAL REGISTRATION NUMBERS

SIMPLIFY‐1 (NCT01969838) and SIMPLIFY‐2 (NCT02101268).

## Supporting information


Supporting information S1.

## Data Availability

Sierra Oncology, a GSK company, commits to share clinical study data with qualified researchers to enable enhancement of public health. As such, Sierra will share anonymized patient‐level data on request or if required by law or regulation. Qualified scientific and medical researchers can request patient‐level data for studies of Sierra pharmaceutical substances listed on ClinicalTrials.gov and approved by health authorities in the United States and the EU. Patient‐level data for studies of newly approved pharmaceutical substances or indications can be requested nine months after US Food and Drug Administration and European Medicines Agency approvals. Such requests are assessed at Sierra's discretion, and the decisions depend on the scientific merit of the proposed request, data availability, and the purpose of the proposal. If Sierra agrees to share clinical data for research purposes, the applicant is required to sign an agreement for data sharing before data release, to ensure that the patient data are de‐identified. In case of any risk of re‐identification on anonymized data despite measures to protect patient confidentiality, the data will not be shared. The patients’ informed consent will always be respected. If the anonymization process provides futile data, Sierra will have the right to refuse the request. Sierra will provide access to patient‐level clinical trial analysis datasets in a secured environment upon execution of the data‐sharing agreement. Sierra will also provide the protocol, statistical analysis plan, and the clinical study report synopsis, if needed. For additional information or requests for access to Sierra clinical trial data for research purposes, please contact us at GSKClinicalSupportHD@gsk.com
