# Supplementary material for: Symptomatic benefit of momelotinib in patients with myelofibrosis: Results from the SIMPLIFY phase III studies
Source: Cancer Med. 2023 Apr 6;12(9):10612–24. doi: 10.1002/cam4.5799 (PMC10225216; doi:10.1002/cam4.5799)

**Supporting Material**

**Supporting Tables**

TABLE S1 SIMPLIFY-1 Study demographics and baseline characteristics

| **SIMPLIFY-1** | **Overall population** | | | **Symptomatic population  (baseline MPN-SAF TSS ≥10)** | | |
| --- | --- | --- | --- | --- | --- | --- |
|  | **MMB**  **(*n* = 215)**^†^ | **RUX**  **(*n* = 217)**^†^ | **Total**  **(*N* = 432)**^†^ | **MMB**  **(*n* = 145)**^†^ | **RUX**  **(*n* = 147)**^†^ | **Total**  **(*N* = 292)**^†^ |
| **Age** | | | | | | |
| Mean (SD) | 65.0 (10.7) | 64.4 (10.6) | 64.7 (10.6) | 64.5 (10.2) | 64.4 (10.2) | 64.4 (10.2) |
| Median | 67.0 | 66.0 | 66.0 | 65.0 | 66.0 | 65.0 |
| Q1, Q3 | 59.0, 72.0 | 59.0, 71.0 | 59.0, 72.0 | 59.0, 71.0 | 59.0, 72.0 | 59.0, 72.0 |
| Min, max | 28.0, 85.0 | 25.0, 86.0 | 25.0, 86.0 | 28.0, 85.0 | 35.0, 86.0 | 28.0, 86.0 |
| **Age group (years**), **n (%)** | | | | | | |
| <65 | 90 (41.9) | 95 (43.8) | 185 (42.8) | 68 (46.9) | 68 (46.3) | 136 (46.6) |
| ≥65 | 125 (58.1) | 122 (56.2) | 247 (57.2) | 77 (53.1) | 79 (53.7) | 156 (53.4) |
| **Sex at birth, n (%)** | | | | | | |
| Male | 124 (57.7) | 120 (55.3) | 244 (56.5) | 82 (56.6) | 74 (50.3) | 156 (53.4) |
| Female | 91 (42.3) | 97 (44.7) | 188 (43.5) | 63 (43.5) | 73 (49.7) | 136 (46.6) |
| **Race, n (%)** | | | | | | |
| White | 179 (83.3) | 178 (82.0) | 357 (82.6) | 126 (86.9) | 126 (85.7) | 252 (86.3) |
| Black or African American | 2 (0.9) | 2 (0.9) | 4 (0.9) | 1 (0.7) | 2 (1.4) | 3 (1.0) |
| Asian | 17 (7.9) | 20 (9.2) | 37 (8.) | 8 (5.5) | 7 (4.8) | 15 (5.1) |
| Not permitted to be recorded | 15 (7.0) | 16 (7.4) | 31 (7.2%) | 9 (6.2) | 11 (7.5) | 20 (6.8) |
| Other | 2 (0.9) | 1 (0.5) | 3 (0.7) | 1 (0.7) | 1 (0.7) | 2 (0.7) |
| **MF disease type, n (%)** | | | | | | |
| Primary MF | 128 (59.5) | 116 (53.5) | 244 (56.5) | 86 (59.3) | 80 (54.4) | 166 (56.8) |
| Post-polycythemia vera MF | 48 (22.3) | 50 (23.0) | 98 (22.7) | 32 (22.1) | 32 (21.8) | 64 (21.9) |
| Post-essential thrombocythemia MF | 39 (18.1) | 51 (23.5) | 90 (20.8) | 27 (18.6) | 35 (23.8) | 62 (21.2) |
| **International Prognostic Scoring System, n (%)** | | | | | | |
| Intermediate-1 | 46 (21.4) | 43 (19.8) | 89 (20.6) | 30 (20.7) | 30 (20.4) | 60 (20.5) |
| Intermediate-2 | 76 (35.3) | 67 (30.9) | 143 (33.1) | 51 (35.2) | 50 (34.0) | 101 (34.6) |
| High | 93 (43.3) | 107 (49.3) | 200 (46.3) | 64 (44.1) | 67 (45.6) | 131 (44.9) |
| **TSS at baseline** | | | | | | |
| *N* | 213 | 214 | 427 | 145 | 147 | 292 |
| Mean (SD) | 19.4 (13.2) | 17.9 (11.5) | 18.7 (12.4) | 25.3 (10.8) | 23.4 (9.5) | 24.3 (10.2) |
| Median | 17.4 | 16.4 | 17.0 | 23.5 | 21.9 | 23.0 |
| Q1, Q3 | 8.4, 27.6 | 8.6, 25.0 | 8.6, 26.4 | 16.2, 32.0 | 15.5, 28.1 | 16.0, 30.1 |
| Min, max | 0, 52.9 | 0, 56 | 0, 55.7 | 10.0, 52.9 | 10.1, 55.7 | 10.0, 55.7 |

Abbreviations: MF, myelofibrosis; MMB, momelotinib; MPN-SAF, Myeloproliferative Neoplasm Symptom Assessment Form; Q, quarter; RUX, ruxolitinib; SD, standard deviation; TSS, total symptom score.
^†^Unless otherwise noted.

TABLE S2 SIMPLIFY-2 Study demographics and baseline characteristics

| **SIMPLIFY-2** | **Overall population** | | | **Symptomatic population  (baseline MPN-SAF TSS ≥10)** | | |
| --- | --- | --- | --- | --- | --- | --- |
|  | **MMB**  **(*n* = 104)**^†^ | **BAT**  **(*n* = 52)**^†^ | **Total**  **(*N* = 156)**^†^ | **MMB**  **(*n* = 72)** | **BAT**  **(*n* = 33)** | **Total**  **(*N* = 105)** |
| **Age** | | | | | | |
| Mean (SD) | 66.4 (8.1) | 69.4 (7.4) | 67.4 (8.0) | 65.8 (8.1) | 69.8 (7.0) | 67.0 (8.0) |
| Median | 67.0 | 69.5 | 68.0 | 66.0 | 69.0 | 67.0 |
| Q1, Q3 | 61.5, 72.0 | 64.0, 75.0 | 62.0, 73.5 | 60.5, 71.5 | 64.0, 76.0 | 45.0, 92.0 |
| Min, max | 41, 92 | 52, 82 | 41, 92 | 45.0, 92.0 | 59.0, 82.0 | 62.0, 72.0 |
| **Age group (years), n (%)** | | | | | | |
| <65 | 41 (39.4) | 14 (26.9) | 55 (35.3) | 32 (44.4) | 9 (27.3) | 41 (39.0) |
| ≥65 | 63 (60.6) | 38 (73.1) | 101 (64.7) | 40 (55.6) | 24 (72.7) | 64 (61.0) |
| **Sex at birth, n (%)** | | | | | | |
| Male | 69 (66.3) | 24 (46.2) | 93 (59.6) | 44 (61.1) | 9 (27.3) | 53 (51.0) |
| Female | 35 (33.7) | 28 (53.8) | 63 (40.4) | 28 (38.9) | 24 (72.7) | 52 (49.5) |
| **Race, n (%)** | | | | | | |
| White | 83 (79.8) | 44 (84.6) | 127 (81.4) | 61 (84.7) | 29 (87.9) | 90 (85.7) |
| Black or African American | 6 (5.8) | 0 | 6 (3.8) | 3 (4.2) | 0 (0.0) | 3 (2.9) |
| Not permitted | 15 (14.4) | 8 (15.4) | 23 (14.7) | 8 (11.1) | 4 (12.1) | 12 (11.4) |
| **MF disease type, n (%)** | | | | | | |
| Primary MF | 64 (61.5) | 30 (57.7) | 94 (60.3) | 43 (59.7) | 17 (51.5) | 60 (57.1) |
| Post-polycythemia vera MF | 18 (17.3) | 12 (23.1) | 30 (19.2) | 15 (20.8) | 9 (27.3) | 24 (22.9) |
| Post-essential thrombocythemia MF | 22 (21.2) | 10 (19.2) | 32 (20.5) | 14 (19.4) | 7 (21.2) | 21 (20.0) |
| **Dynamic International Prognostic Scoring System, n (%)** | | | | | | |
| Intermediate-1 | 23 (22.1) | 16 (30.8) | 39 (25.0) | 18 (25.0) | 8 (24.2) | 26 (24.8) |
| Intermediate-2 | 62 (59.6) | 28 (53.8) | 90 (57.7) | 39 (54.2) | 19 (57.6) | 58 (55.2) |
| High | 19 (18.3) | 8 (15.4) | 27 (17.3) | 15 (20.8) | 6 (18.2) | 21 (20.0) |
| **TSS at baseline** | | | | | | |
| *N* | 104 | 52 | 156 | 72 | 33 | 105 |
| Mean (SD) | 18.5 (13.0) | 20.5 (16.0) | 19.2 (14.0) | 23.4 (11.5) | 27.7 (13.7) | 24.7 (12.4) |
| Median | 15.6 | 15.9 | 15.6 | 21.6 | 24.1 | 22.4 |
| Q1, Q3 | 8.7, 25.8 | 7.1, 29.9 | 8.5, 27.0 | 14.2, 29.6 | 15.3, 37.3 | 14.7, 31.0 |
| Min, max | 0, 56.7 | 0, 55.5 | 0, 56.7 | 10.0, 56.7 | 10.0, 55.5 | 10.0, 56.7 |

Abbreviations: BAT, best available therapy; MF, myelofibrosis; MMB, momelotinib; MPN-SAF, Myeloproliferative Neoplasm Symptom Assessment Form; Q, quarter; SD, standard deviation; TSS, total symptom score.

**^a^**Unless otherwise noted.

**Supporting Figures**

FIGURE S1 Cumulative distribution function of absolute change in MPN-SAF TSS from baseline to week 24 in the A) SIMPLIFY-1 overall population B) SIMPLIFY-2 overall population. BAT, best available therapy; MPN-SAF, Myeloproliferative Neoplasm Symptom Assessment Form; TSS, total symptom score.

**(A)**


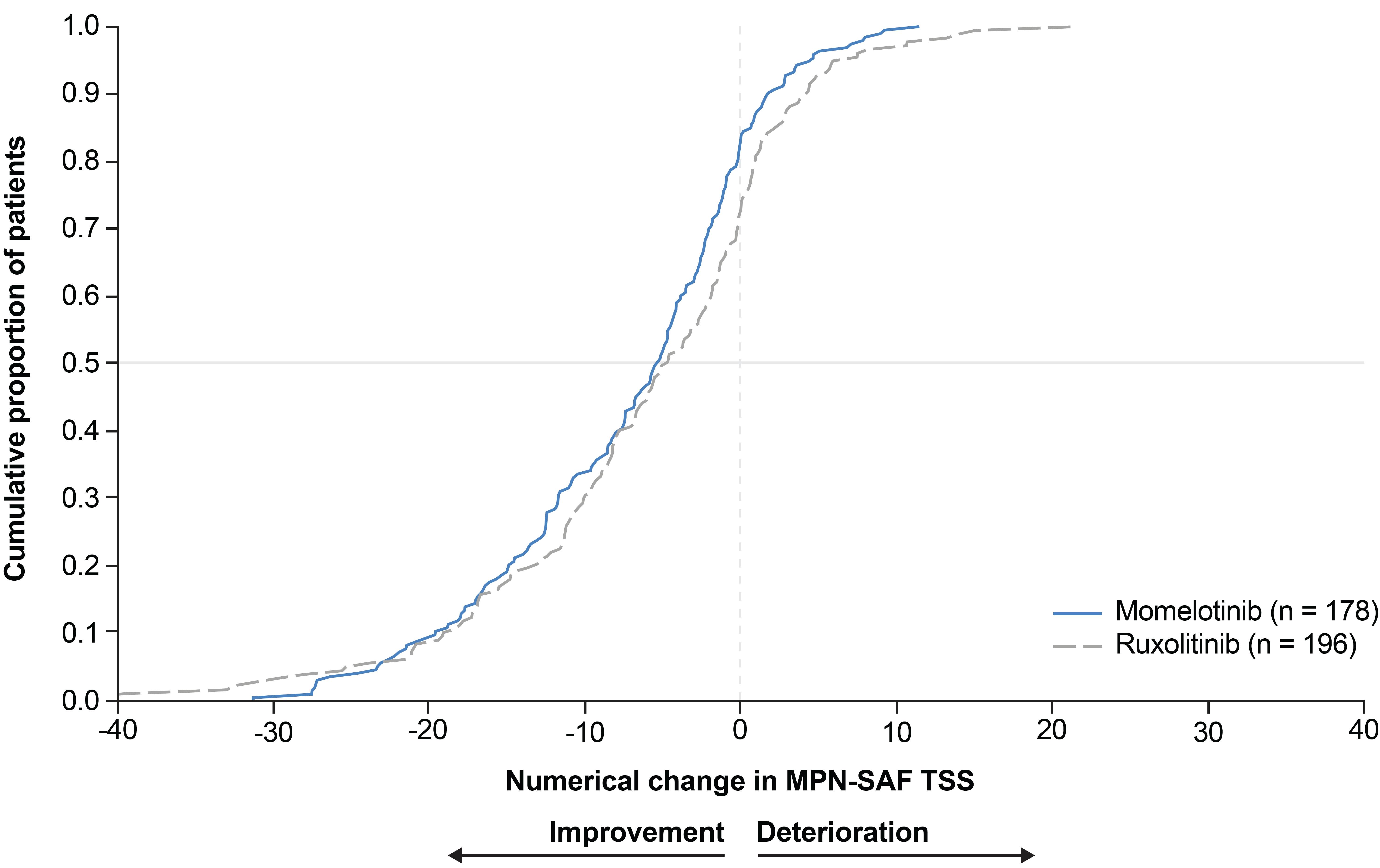


**(B)**


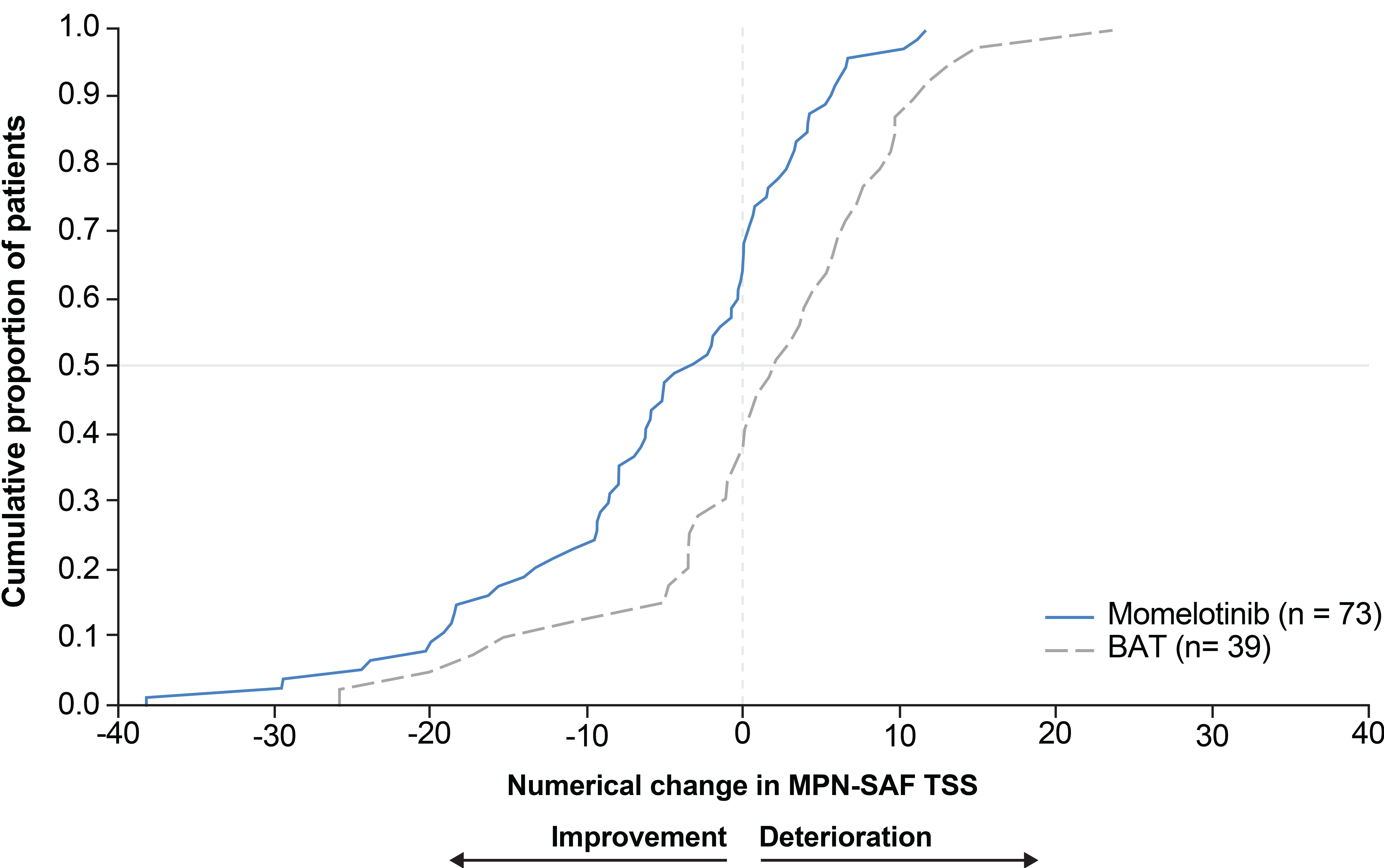


FIGURE S2 MPN-SAF TSS Item distribution at baseline in the overall population of A) SIMPLIFY-1 and B) SIMPLIFY-2. MMB, momelotinib; MPN-SAF, Myeloproliferative Neoplasm Symptom Assessment Form; RUX, ruxolitinib; TSS, total symptom score.

**(A)**


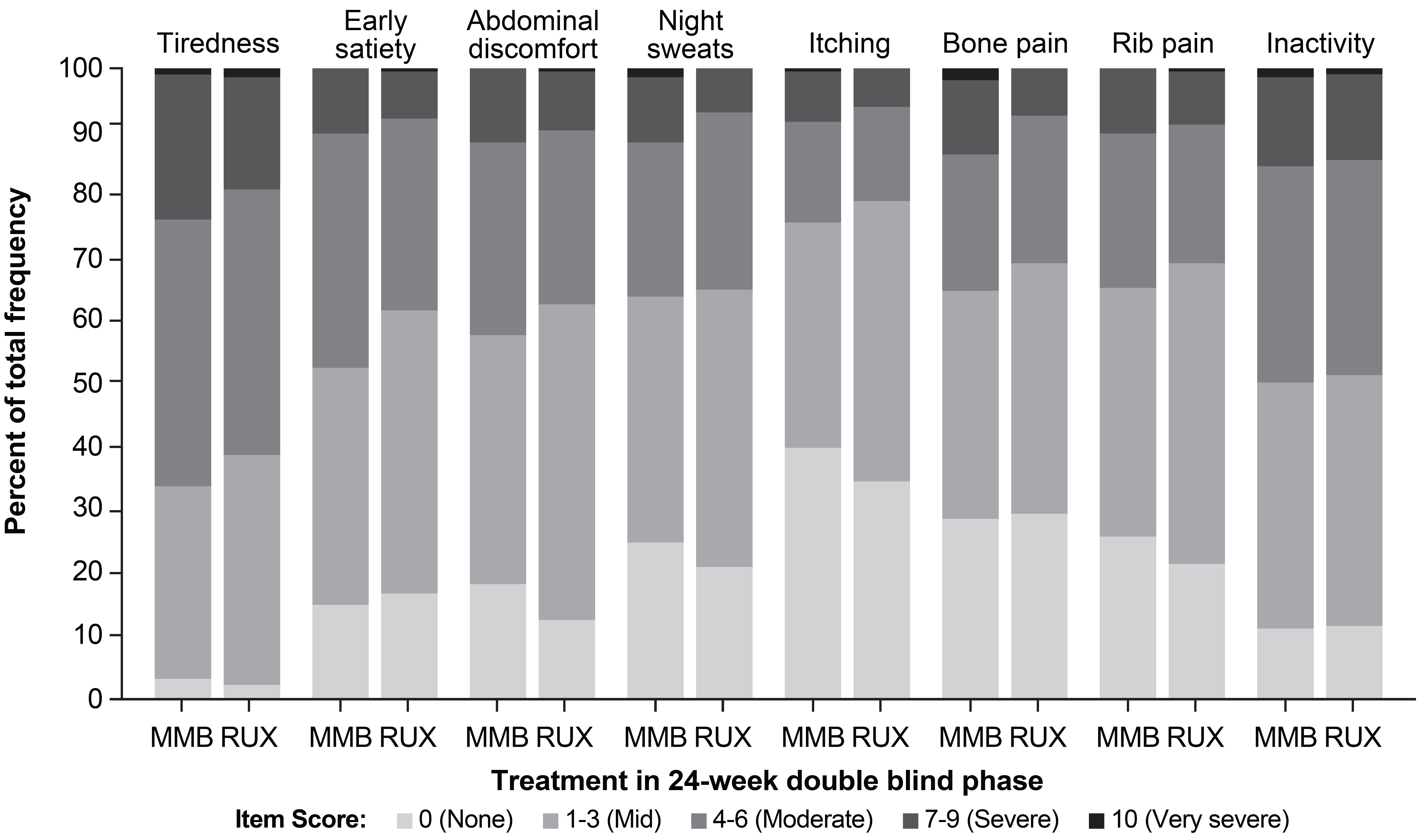


**(B)**


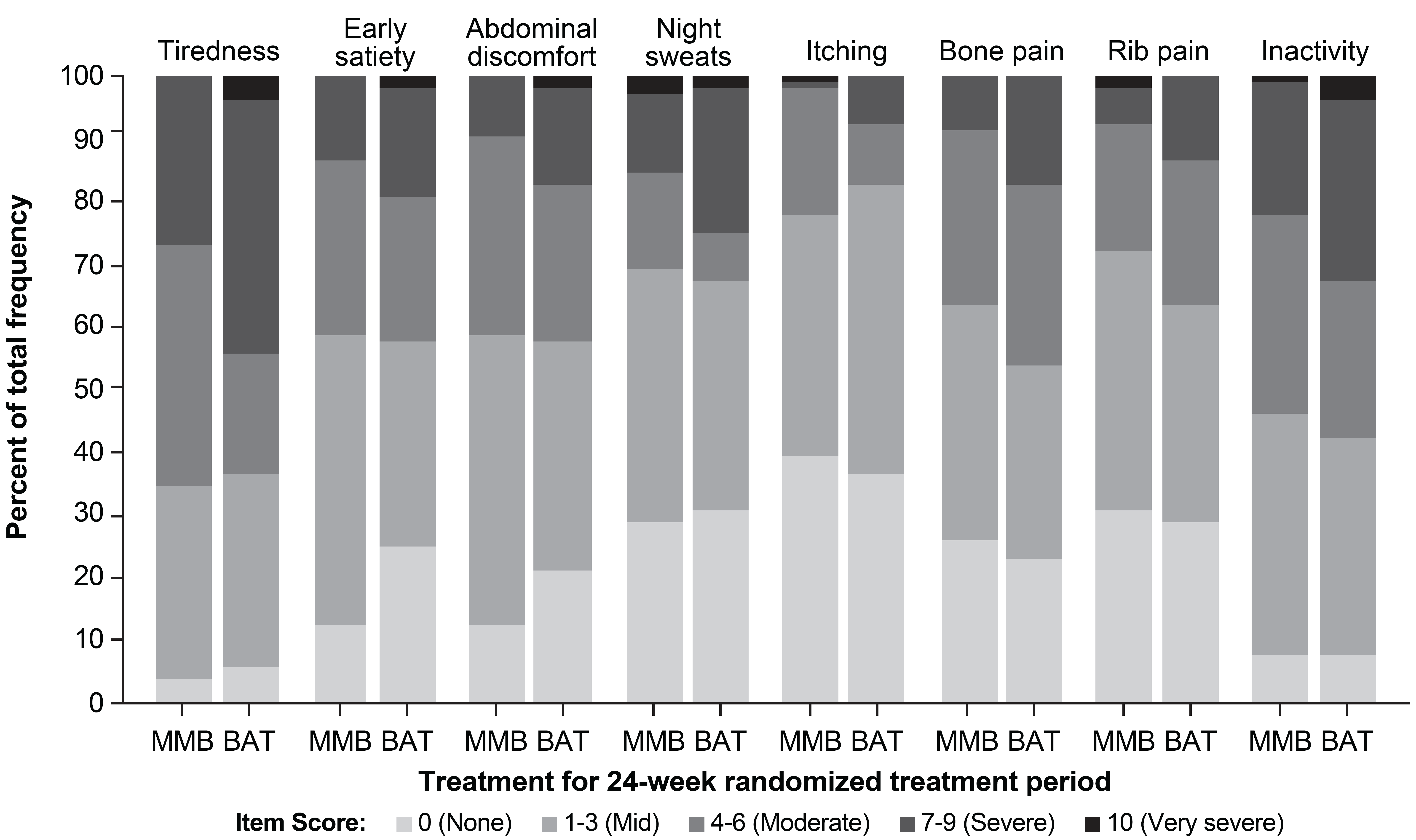

Supplement: Supplementary file 1 — Supporting information S1. [file CAM4-12-10612-s001.docx]
